# Hemolytic uremic syndrome caused by sea anemone sting: a case report

**DOI:** 10.1186/s12882-020-02218-5

**Published:** 2021-01-07

**Authors:** A Young Kim, Kyu Hyang Cho, Seok Hui Kang, Jong Won Park, Jun Young Do, Min Kyoung Kim

**Affiliations:** 1 Division of Nephrology, Department of Internal Medicine, Yeungnam University College of Medicine, 170 Hyeonchung-ro, Nam-gu, 42415 Daegu, Republic of Korea; 2 Division of Hemato-Oncology, Department of Internal Medicine, Yeungnam University College of Medicine, Daegu, Korea

**Keywords:** Acute renal failure, Hemolytic uremic syndrome, Sea anemone, Thrombotic microangiopathy, Case report

## Abstract

**Background:**

Some sea anemone toxins cause renal injuries resembling hemolytic uremic syndrome (HUS). To date, only a few cases of HUS caused by sea anemone stings have been reported. In this case report, we have described an HUS case caused by a sea anemone sting.

**Case presentation:**

In November 2019, a 37-year-old man with no underlying disease was admitted to our hospital. He presented with intense pain, a rash on, and swelling in his right thigh. Two days prior, he had been stung by a sea anemone while scuba diving in Cebu, Philippines. His blood tests revealed renal dysfunction, and his platelet count was normal. However, on day three, the platelet count decreased rapidly. His blood haptoglobin level decreased, and schistocytes were identified on the peripheral blood smear. We suspected thrombotic microangiopathy and started the conventional treatment, comprising hemodialysis, blood transfusion, and antibiotic administration. ADAMTS-13 and genetic test results associated with atypical HUS were normal. Therefore, the patient was diagnosed with HUS caused by a sea anemone toxin.

**Conclusions:**

HUS caused by a sea anemone toxin is rare, but it is a serious medical disease. Clinicians should consider HUS in patients with such clinical presentations, and they should make prompt treatment-related decisions.

## Background

Most sea anemones are harmless to humans [1], but a few of them are highly toxic. The toxin occasionally causes dermatitis and allergic shock, but it seldom causes multiple organ failure [2], hemolysis, and renal injuries, indicating hemolytic uremic syndrome (HUS) [1]. *Phyllodiscus semoni* can cause acute renal failure [2]. Sea anemone stings also cause acute kidney injury with tubular necrosis and severe dermatitis [2]; additionally, re-examination of the kidney biopsy samples revealed endothelial damage in afferent and/or efferent arterioles [3]. HUS is also characterized by hemolytic anemia, thrombocytopenia, and renal impairment [4]. It can be triggered by various factors, such as infection, cancer, organ transplantation, pregnancy, and drugs [5–7]. Since HUS is associated with end-stage renal failure and high mortality rate, its prognosis is poor [8]. Till date, only a few cases of HUS caused by sea anemone stings have been reported. In this case report, we have described a HUS case caused by sea anemone sting.

## Case presentation

In November 2019, a 37-year-old man with no underlying disease presented with pain, a rash, and swelling in the right thigh. Two days prior, he was stung by a sea anemone while scuba diving in Cebu, Philippines. He was administered a tetanus injection at a hospital in Philippines. We made the diagnosis based on the characteristic shape of the wounds, which were caused by a sea anemone tentacle, and the patient’s statement.

**Fig. 1:**
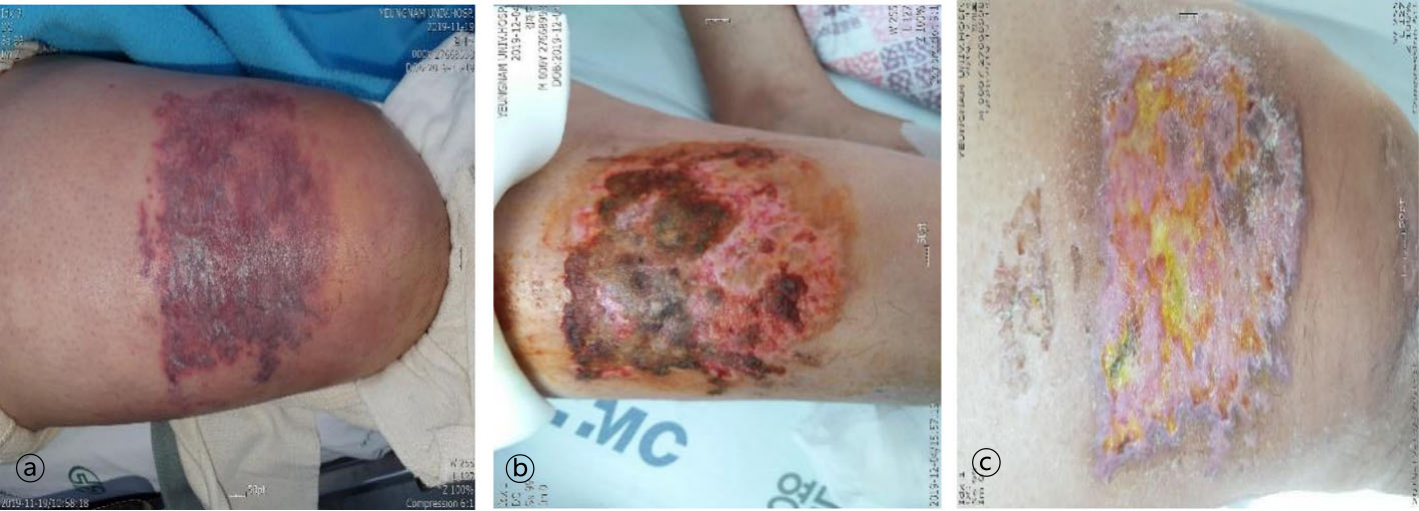
Wound on the right thigh (**a**) on the day of admission, (**b**) at week 1, and (**c**) at week 3

On admission, generalized swelling and redness were observed on the right leg. A 20 × 15 cm wound and a purpuric, reticulated patch were observed on the right thigh (Fig. 1a). His blood pressure was 100/60 mmHg, and his body temperature was 36.6 ℃. His white blood cell count was 17,900 cells/mm^3^, hemoglobin level was 13.9 g/dL, platelet count was 303,000/mm^3^, blood urea nitrogen (BUN) level was 51 mg/dL, creatinine level was 5.12 mg/dL, aspartate aminotransferase level was 370 IU/L, alanine aminotransferase level was 209 IU/L, and serum creatine phosphokinase level was 401 IU/L (1-171 IU/L). Urine blood was + 2 positive, and RBC count was 0–1/HPF. Rhabdomyolysis was suspected, and subsequently, fluid therapy was initiated.

On day three, his platelet count rapidly decreased (36,000 cells/mm^3^); and renal dysfunction (BUN and creatinine concentrations were 85 and 9.86 mg/dL, respectively) gradually worsened. Oliguria was confirmed along with worsening pulmonary edema. Therefore, we initiated conventional hemodialysis.

On day four, his body temperature was 38.4 ℃ and C-reactive protein (CRP) concentration was 5.097 mg/dL. We suspected sepsis due to cellulitis and initiated broad-spectrum antibiotics. His white blood cell count was 10,760 cells/mm^3^, and leukocytosis was better than that at the time of the emergency visit. Fever was observed only once (38.4 ℃), and subsequently, his body temperature was normal. His blood pressure was also stable. Blood culture test results were negative; therefore, cellulitis was confirmed without diagnosis of sepsis. We also excluded disseminated intravascular coagulation since the results in our case fulfilled only two of the four Korean Society of Thrombosis and Hemostasis disseminated intravascular coagulation (DIC) criteria. DIC can be diagnosed when three or more of the following diagnostic criteria are satisfied: (1) Positive fibrin degradation products or D-dimer, (2) Platelet count < 100,000 cells/mm^3^, (3) Fibrinogen concentration < 150 mg/dL, and (4) prothrombin time (PT) ≥ 3 sec or activated partial thromboplastin time (aPTT) ≥ 5 sec [9]. In our case, the D-dimer and fibrinogen concentrations were 5.48 µg/mL (0-0.5 µg/mL) and 542 mg/dL, respectively, and PT and aPTT were 12 sec (0.4–13.3 sec) and 29.6 sec (23.2–39.4 sec), respectively.

The hemoglobin levels decreased to 7.4 g/dL with progressive thrombocytopenia and renal dysfunction. His blood lactate dehydrogenase (LDH) level was 3,354 IU/L (150–550 IU/L), and his haptoglobin levels decreased to 10 mg/dL (50–320 mg/dL). Schistocytes were observed on the peripheral blood (PB) smear, and the patient complained of a mild headache. We suspected thrombotic microangiopathy syndrome based on microangiopathic hemolytic anemia, thrombocytopenia, and renal dysfunction. Thrombotic thrombocytopenic purpura (TTP) could not be ruled out until ADAMTS-13 results were available. Therefore, we performed a plasma exchange until ADAMTS-13 results were obtained. Complement sampling was performed prior to plasma exchange. The concentrations of serum complement 3 and complement 4 were 109.9 mg/dL (90–180 mg/dL) and 35.6 mg/dL (10–40 mg/dL), respectively, antinuclear antibody was negative, concentrations of antineutrophil cytoplasmic antibody was 0.21 U/mL (≤ 0.9 U/mL), anti-glomerular basement membrane antibody was 0.8 U/mL (< 20.0 U/mL), and ADAMTS-13 was 46.9%. Since TTP was excluded based on normal ADAMTS-13 level, we discontinued plasma exchange. After discontinuation of plasma exchange, the platelet count began to increase. The genetic test results associated with atypical HUS were normal. Genetic testing was performed at the Department of Laboratory Medicine, Samsung Seoul Hospital. A total of 18 genes (*C3*, *C4BPA*, *C4BPB*, *CD46*, *CFB*, complement factor H (*CFH*), *CFHR1*, *CFHR2*, *CFHR3*, *CFHR4*, *CFHR5*, *CFI*, *DGKE*, *LMNA*, *THBD*, *MMACHC*, *PLG*, *ADAMTS-13*) were tested. No clinically significant variants were detected in any of the 18 genes, including the CFH gene that accounts for about 22% of atypical HUS. Based on the above results, the patient was diagnosed with HUS caused by a sea anemone toxin. We continued conventional treatment that included hemodialysis, blood transfusion, and antibiotic administration. The wound turned necrotic, and it was debrided and dressed with betadine-soaked dressings (Fig. 1b).

On day 10, the hemoglobin level and platelet count normalized. In addition, the urine volume gradually increased, and hemodialysis was discontinued. The serum creatinine level was 7.2 mg/dL, LDH level decreased, and haptoglobin level normalized.

**Fig. 2:**
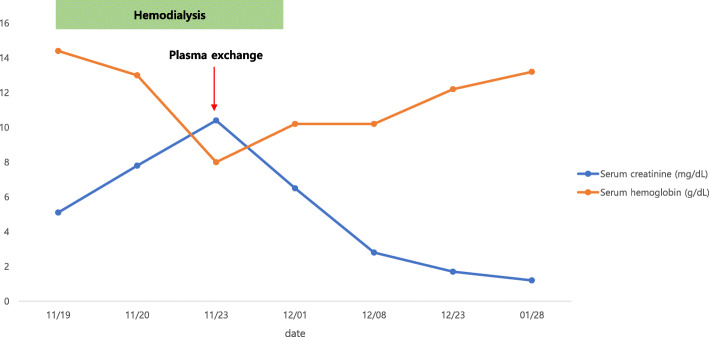
Serum hemoglobin and creatinine levels at different time points

**Fig. 3:**
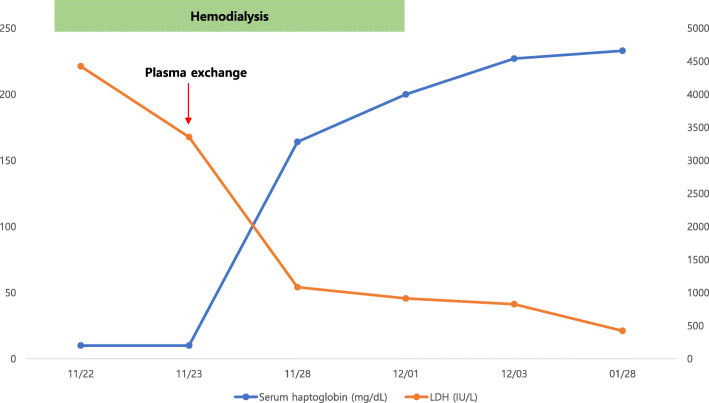
Serum lactate dehydrogenase (LDH) and haptoglobin levels at different time points

**Fig. 4:**
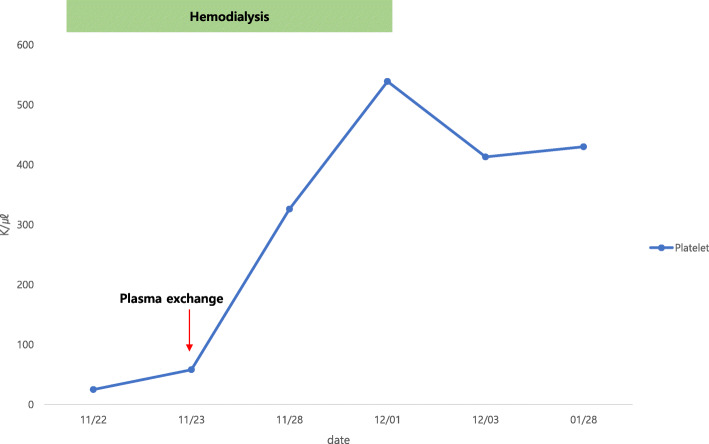
Serum platelet count at different time points

On day 21, the CRP level was 0.874 mg/dL. Therefore, we discontinued antibiotic administration. The wound was healing (Fig. 1c), and there was no pain or swelling on the right thigh. Even after discontinuing hemodialysis, the serum creatinine level continued to decrease and serum hemoglobin level remained stable (Fig. 2). Additionally, the serum haptoglobin level, LDH level, platelet count, and schistocyte count on the PB smear were stable (Figs. 3 and 4). We continued wound dressing, blood tests and kept him under close observation. Finally, he was discharged on day 28. Six months after discharge, his serum creatinine level, hemoglobin level, and platelet count were normal.

## Discussion and conclusions

The severity of sea anemone sting depends on the amount of venom, nature of toxicity, size of the sting relative to the surface area of the victim, and victim’s general health [10]. In animal studies, the venom of *Phyllodiscus semoni*, a family of sea anemones, induces acute renal injury [1]. In a rat model, sea anemone venom binds directly to the glomeruli, and it acts as a nephrotoxin [1]. It inhibits the expression of C regulators (CD 55, CD 59) and causes accumulation of complement activation products (C3b, C5b-9) [1]. It is suggested that complement activation may cause acute renal injury after a sea anemone sting [1]. In a rodent model of thrombotic microangiopathy (TMA) caused by sea anemone toxin, effectiveness of anti-complement therapy was reported [11]. Treatment with soluble CR1 (complement activation inhibitor) suppressed sea anemone toxin-induced glomerular damage and renal tubular epithelial damage [11]. The pathology of renal injuries observed in a rat model resembled that of the acute phase of HUS [1]. Renal injury is confirmed not only by the endothelial injury of the glomerulus but also by glomerular epithelial cells [1]. Since the platelet count was low and prone position was not feasible due to the leg wound, we did not perform renal biopsy. Since end-stage renal failure occurs in a few patients after apparent recovery, renal biopsies are not recommended to determine prognosis [12, 13]. Thus, it has more risks than benefits.

Previous studies have reported acute renal failure or fulminant hepatic failure caused by a sea anemone sting [14]. However, to the best of our knowledge, this is the first case report of HUS in a human caused by sea anemone sting. The laboratory results revealed hemolytic anemia, thrombocytopenia, schistocytes on the PB smear, and renal failure. Autoimmune antibody, ADAMTS-13, and genetic test results associated with atypical HUS were normal. Therefore, the patient was diagnosed with HUS caused by a sea anemone toxin.

Treatment of secondary HUS comprises withdrawal of the triggering factor and supportive care [15]. Plasma exchange is empirically used when TTP cannot be excluded [16]. Supportive treatment has led to a decline in the acute mortality rate from > 30% before 1970 [17] to the current rate of < 5% [18]. Although rapid plasma exchange and conservative treatments, such as dialysis, were performed only once, they could reduce the risk of mortality. The patient demonstrated good recovery, and he had no complications on discharge. Duration of anuria is an important predictor of chronic kidney disease. Therefore, patients who are anuric in the acute phase should be closely monitored for several years [14]. We will continue to observe the patient’s general condition, including urinary protein excretion, hypertension, and increase in serum creatinine level, through the outpatient department.

Although evidence exists on the direct nephrotoxicity caused by the toxin and increasing acute mortality rate, the underlying mechanisms of renal injury in humans are not clearly understood. Presently, complement inhibitors (anti-C5 antibody, Eculizumab) used for treatment of atypical HUS in human [1]. After considering the pathophysiology of HUS in a rat model, anti-complement therapy might be an option to treat HUS caused by sea anemone toxin.

HUS caused by a sea anemone toxin in humans is rare, but it is a serious medical disease. Through this report, clinicians should consider HUS in patients with such presentations, and they should make prompt treatment-related decisions.

## Data Availability

All relevant data are present in the case report.

## Abbreviations

HUS: Hemolytic uremic syndrome
BUN: Blood urea nitrogen
DIC: Disseminated intravascular coagulation
PT: Prothrombin time
aPTT: Activated partial thromboplastin time
CRP: C-reactive protein
LDH: lactate dehydrogenase
PB: peripheral blood
TTP: Thrombotic thrombocytopenic purpura
CFH: complement factor H
TMA: Thrombotic microangiopathy
