# Correction to: Hemolytic uremic syndrome caused by sea anemone sting: a case report

**DOI:** 10.1186/s12882-021-02446-3

**Published:** 2021-07-07

**Authors:** A. Young Kim, Kyu Hyang Cho, Seok Hui Kang, Jong Won Park, Jun Young Do, Min Kyoung Kim

**Affiliations:** 1 Division of Nephrology, Department of Internal Medicine, Yeungnam University College of Medicine, 170 Hyeonchung-ro, Nam-gu, 42415 Daegu, Republic of Korea; 2 Division of Hemato-Oncology, Department of Internal Medicine, Yeungnam University College of Medicine, Daegu, Korea

**Correction to: BMC Nephrol (2021) 22:14**

**https://doi.org/10.1186/s12882-020-02218-5**

Following publication of the original article [1], the authors identified an error in the affiliation of author Jun Young Do and author Min Kyoung Kim.

The affiliations have been updated above and the original article has been corrected.
